# Nocturnal BiPAP Therapy Improved Chronic Respiratory Failure in Chilaiditi Syndrome

**DOI:** 10.1002/jgf2.70102

**Published:** 2026-02-15

**Authors:** Takeshi Kouga, Reizo Tsukamoto

**Affiliations:** ^1^ Department of General Medicine Chigasaki Tokusyukai Hospital Kanagawa Japan

**Keywords:** BiPAP therapy, Chilaiditi syndrome, chronic respiratory failure, COPD

## Abstract

**Background:**

Chilaiditi syndrome includes the Chilaiditi sign, a radiological finding where a segment of bowel loop or small intestine has wandered between the liver and diaphragm and also any symptoms related to intestinal interference.

**Case Presentation:**

We report the case of a 79‐year‐old man with Chilaiditi syndrome whose dyspnea and sleep disturbance improved after nocturnal biphasic positive airway pressure (BiPAP) therapy implementation.

**Discussion:**

BiPAP therapy may contribute to thoracic pressure elevation and reduce diaphragmatic compression due to intestinal enlargement, thus leading to improvements in respiratory symptoms.

**Conclusion:**

BiPAP therapy should therefore be considered as a treatment option for Chilaiditi syndrome patients.

## 1 Background

The Chilaiditi sign is a radiological finding where a segment of bowel loop or small intestine has wandered between the liver and diaphragm. This occurs more frequently in males than in females (ratio of 4:1) and appears in approximately 0.02%–0.14% of various radiological studies [1]. Chilaiditi syndrome, which includes the Chilaiditi sign and any symptoms related to intestinal interference, was first defined in 1910 by Demetrius Chilaiditi, a Greek radiologist.

Chilaiditi syndrome can be triggered by several pathological conditions, such as congenital malposition or small liver due to cirrhosis or hepatectomy. Chronic obstructive lung disease (COPD) patients can also develop Chilaiditi syndrome due to enlargement of the lower thoracic cavity, which can increase the risk of intestinal invagination [2, 3]. The symptoms of Chilaiditi syndrome can vary and include abdominal pain, nausea, flatulence, and dyspnea.

To date, there is no widely accepted standard management for Chilaiditi syndrome [4]. Treatment is usually conservative and can include nasogastric bowel decompression. Surgical intervention may be considered in severe cases. Here, we report the case of a patient with Chilaiditi syndrome whose dyspnea and sleep disturbance improved after nocturnal biphasic positive airway pressure (BiPAP) therapy.

## 2 Case Presentation

A 79‐year‐old man visited our hospital complaining of shortness of breath on exertion and sleep disturbance. His symptoms gradually worsened, especially overnight. He was previously diagnosed with COPD and underwent colon cancer surgery 14 years ago. He had been on antihypertensive and statin medications but had not been treated for COPD.

During the first hospital visit, physical examination revealed mild anasarca and markedly diminished breath sounds on the right side. The patient's percutaneous oxygen saturation (SpO_2_) was 93% at rest in ambient air but quickly dropped to 80% while walking and he also developed severe dyspnea. Chest X‐rays showed right diaphragm elevation as well as colon gas under the right diaphragm. The right diaphragmatic movement was limited to a few centimeters on deep breathing but was not unusual. Based on these findings, he was diagnosed with chronic respiratory failure due to Chilaiditi syndrome.

The following day, the patient underwent a pulmonary function test with the following results: forced vital capacity (FVC) was 1.5 L, vital capacity percentage was 57.0%, forced expiratory volume in one second was 74.7%, and flow at 50%/flow at 25% of FVC was 4.6, indicating moderate restrictive lung function and small airway obstruction. A modified sleep study that was conducted to examine the reasons for the sleep disturbance showed severe hypoxia during sleep where the lowest SpO_2_ was 45%, while the rate of total time less than 90% SpO_2_ was 67.5% of total sleep time. The respiratory disturbance index (RDI; average number of apnea and hypopnea events every hour, normal range < 5) was 13.6, suggesting the existence of mild sleep apnea. In echocardiography, the ejection fraction (EF) was 74.0% and estimated right ventricular systolic pressure (RVSP) was 30 mmHg.

To relieve the patient's dyspnea and sleep disturbance, nocturnal BiPAP therapy was introduced (inspiratory positive airway pressure: 8 cmH_2_O, expiratory positive airway pressure: 4 cmH_2_O). After BiPAP therapy initiation, his shortness of breath on exertion gradually decreased, and his SpO_2_ stayed above 90% even while walking. His edema was also alleviated, and he was able to sleep comfortably during the night.

**FIGURE 1:**
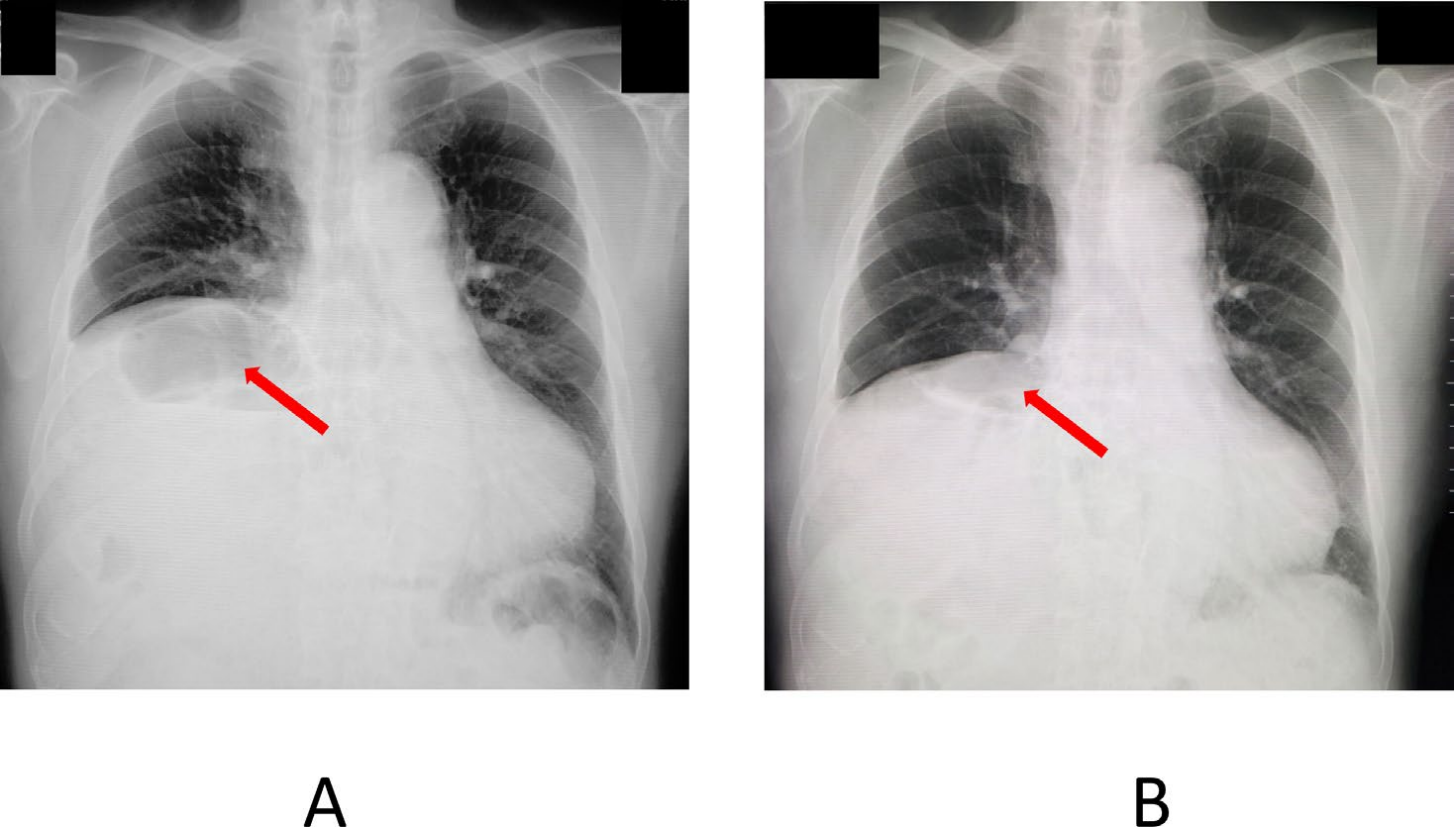
Chest X‐rays before (A) and after (B) introduction of BiPAP therapy. Both images show colonic gas below the right diaphragm (arrows).

Echocardiography findings one year after the initiation of BiPAP therapy were as follows: the EF was 70.0% and estimated RVSP was 45 mmHg, while the daytime venous partial pressure of carbon dioxide (PCO_2_) was 54.5 mmHg in ambient air, indicating the possibility of pulmonary hypertension and sustained hypercapnia (PCO_2_ data before BiPAP therapy was not available). Four years after the initiation of BiPAP therapy, additional pulmonary function tests and chest X‐rays did not show any changes (Table 1, Figure 1). On the other hand, the second modified sleep study showed fine improvement in oxygenation, where the lowest SpO_2_ was 77% and the rate of total time less than 90% SpO_2_ was only 11.8% of total sleep time. However, the RDI was slightly worse (20.1). He has continued BiPAP therapy for more than 4 years without any co‐interventions.

**TABLE 1:** Results of pulmonary function tests, echocardiography, and sleep studies before and after BiPAP therapy implementation.

	Before	After
FVC	1.5 L	1.5 L
%VC	57.0%	58.0%
FEV1%	74.7%	70.7%
V50/V25	4.6	5.3
EF	74.0%	70.0%
estimated RVSP	30 mmHg	45 mmHg
RDI	13.6	20.1
Lowest SpO2 during sleep	45%	77%
Rate of total time less than 90% SpO2 during sleep	67.5%	11.8%

Abbreviations: BiPAP, biphasic positive airway pressure; EF, ejection fraction; FEV1%, forced expiratory volume in one second; FVC, forced vital capacity; ODI, oxygen desaturation index; RDI, respiratory disturbance index; RVSP, right ventricular systolic pressure; SpO_2_, percutaneous oxygen saturation; V50/V25, flow at 50%/flow at 25% of FVC; %VC, vital capacity percentage.

## 3 Discussion

In general, Chilaiditi syndrome patients do not present with severe symptoms, but respiratory failure can occasionally occur, likely as a result of limited diaphragmatic excursion due to compression of the intruding intestine under the diaphragm [2]. Similar to our case, dyspnea in Chilaiditi syndrome is usually worse at night while the patient is supine [5].

Nocturnal hypoxemia during sleep has also been reported in COPD patients [6], and COPD itself is a risk factor for Chilaiditi syndrome, since chronic hyperinflation of the chest wall cavity with subsequent elongation of the lower thoracic cage creates a broader space for invasion of the intestine [2]. Thus, dyspnea and sleep disturbance symptoms in our patient may be associated with both Chilaiditi syndrome and COPD.

BiPAP therapy was administered to our patient to protect his respiratory muscle fatigue, leading to improvements in small airway obstruction and diaphragmatic excursion during the night but not during more rigorous daytime activities. In addition to improving the patient's sleep condition, BiPAP therapy alleviated his shortness of breath and enhanced his long‐distance gait, suggesting that the therapy may have carryover effects. However, the underlying mechanism remains to be elucidated. Effects such as gas deflation and relief of respiratory muscle fatigue may be sustained after overnight BiPAP therapy.

In this case report, there are some indications that the BiPAP therapy itself improved the degree of respiratory failure in Chilaiditi syndrome. First, although the patient's sleep condition improved, his RDI worsened slightly after BiPAP therapy initiation, indicating the existence of central sleep apnea. Thus, sleep apnea was not thought to be a dominant pathology for the sleep disturbance. Second, after the initiation of BiPAP therapy, the results of the pulmonary function test did not change, suggesting the existence of sustained hypercapnia and pulmonary hypertension, so BiPAP therapy did not impact the patient's COPD state even after his symptoms were alleviated. His previous anasarca might be derived from the nocturnal cardiac strain related to COPD.

Currently, there is no standard management protocol for Chilaiditi syndrome. Some physicians recommend the use of a management staging system categorized by symptoms and severity [4]. Other reports highlight the effectiveness of non‐invasive ventilation [7] or a high‐flow oxygen [8] approach to relieve acute respiratory distress in Chilaiditi syndrome patients by elevating thoracic pressure. BiPAP therapy may contribute to thoracic pressure elevation and reduce diaphragmatic compression due to intestinal enlargement, thus leading to improvements in respiratory symptoms. In this case, BiPAP therapy itself did not impact the pulmonary function and sleep apnea. However, we believe that it had a positive effect against COPD‐related nocturnal cardiac strain and respiratory muscle fatigue. Thus, BiPAP therapy should be considered as a treatment option for Chilaiditi syndrome patients with COPD.

## 4 Conclusion

To our knowledge, this is the first case where BiPAP therapy was introduced to relieve respiratory symptoms in Chilaiditi syndrome, which is a rare disorder. BiPAP therapy should therefore be considered as a treatment option for Chilaiditi syndrome patients in the future.

## Author Contributions

T.K. was involved in the literature search and drafting of the manuscript. R.T. was involved in the literature search and patient clinical care.

## Funding

No funding was received for this manuscript.

## Ethics Statement

This manuscript conforms to the provisions of the Declaration of Helsinki in 1995.

## Consent

Informed consent was obtained from the patient, and his anonymity was preserved.

## Conflicts of Interest

The authors declare no conflicts of interest.

## Data Availability

The data that support the findings of this study are available from the corresponding author upon reasonable request.
